# The Effects of Kaempferol-Inhibited Autophagy on Osteoclast Formation

**DOI:** 10.3390/ijms19010125

**Published:** 2018-01-02

**Authors:** Chang-Ju Kim, Sang-Hun Shin, Bok-Joo Kim, Chul-Hoon Kim, Jung-Han Kim, Hae-Mi Kang, Bong-Soo Park, In-Ryoung Kim

**Affiliations:** 1 Department of Oral and Maxillofacial Surgery, Pusan National University Dental Hospital, 20, Geumo-ro, Mulgeum-eup, Yangsan-si 50612, Gyeongsangnam-do, Korea; changju75@hanmail.net (C.-J.K.); ssh8080@pusan.ac.kr (S.-H.S.); 2 Department of Oral and Maxillofacial Surgery, Medical center, Dong-A University, 26, Daesingongwon-ro, Seo-gu, Busan 49201, Korea; samehope@naver.com (B.-J.K.); bbp2000@hanmail.net (C.-H.K.); noenemyguy@hanmail.net (J.-H.K.); 3 BK21 PLUS Project, School of Dentistry, Pusan National University, Busandaehak-ro, 49, Mulguem-eup, Yangsan-si 50612, Gyeongsangnam-do, Korea; khaemi90@naver.com (H.-M.K.); parkbs@pusan.ac.kr (B.-S.P.); 4 Department of Oral Anatomy, School of Dentistry, Pusan National University, Busandaehak-ro, 49, Mulguem-eup, Yangsan-si 50612, Gyeongsangnam-do, Korea

**Keywords:** bone remodeling, osteoporosis, bisphosphonate-related osteonecrosis of the jaw (BRONJ), osteoclast differentiation, autophagy, flavonoids

## Abstract

Kaempferol, a flavonoid compound, is derived from the rhizome of *Kaempferia galanga L*., which is used in traditional medicine in Asia. Autophagy has pleiotropic functions that are involved in cell growth, survival, nutrient supply under starvation, defense against pathogens, and antigen presentation. There are many studies dealing with the inhibitory effects of natural flavonoids in bone resorption. However, no studies have explained the relationship between the autophagic and inhibitory processes of osteoclastogenesis by natural flavonoids. The present study was undertaken to investigate the inhibitory effects of osteoclastogenesis through the autophagy inhibition process stimulated by kaempferol in murin macrophage (RAW 264.7) cells. The cytotoxic effect of Kaempferol was investigated by MTT assay. The osteoclast differentiation and autophagic process were confirmed via tartrate-resistant acid phosphatase (TRAP) staining, pit formation assay, western blot, and real-time PCR. Kaempferol controlled the expression of autophagy-related factors and in particular, it strongly inhibited the expression of p62/SQSTM1. In the western blot and real time-PCR analysis, when autophagy was suppressed with the application of 3-Methyladenine (3-MA) only, osteoclast and apoptosis related factors were not significantly affected. However, we found that after cells were treated with kaempferol, these factors inhibited autophagy and activated apoptosis. Therefore, we presume that kaempferol-inhibited autophagy activated apoptosis by degradation of p62/SQSTM1. Further study of the *p62/SQSTM1* gene as a target in the autophagy mechanism, may help to delineate the potential role of kaempferol in the treatment of bone metabolism disorders.

## 1. Introduction

Bone remodeling is a lifelong physiological process through which bone tissue is regenerated [1,2,3]. Mature bone is removed through resorption by osteoclasts and new bone is formed by osteoblasts [3,4]. Osteoporosis is a metabolic bone disorder caused by an imbalance in the skeletal turnover so that bone resorption by osteoclasts exceeds bone formation by osteoblast [5,6]. Bone resorption is a specific function of osteoclasts, which erode the bone surface; these cells are targeted by anti-osteoporosis therapy [6]. Bisphosphonates (BP) are clinically used in the treatment of osteoporosis and bone metastases [7]. BP has been reported in several cases with side effects such as gastrointestinal complaints, pyrexia and hypocalcemia, and especially bisphosphonate-related osteonecrosis of the jaw (BRONJ) which is a well-documented devastating caused by long-term use of BP [8,9]. During the process of osteoclastogenesis, the final differentiated cells are characterized as tartrate-resistant acid phosphatase (TRAP)-positive cells and multi-nucleated cells, and these two aspects are widely regarded as biological markers of mature osteoclasts [10]. Osteoclasts are derived from hematopoietic progenitors of the monocyte-macrophage [11]. Osteoclast formation, activation, and survival are principally regulated by macrophage colony-stimulating factor (MCSF), receptor activators of nuclear κB (RANK), RANK ligand (RANKL), and osteoprotegerin (OPG) [12]. In the presence of receptor activators of nuclear factor-κB ligand (RANKL), mouse RAW 264.7 cells possess the ability to differentiate into multinucleated, mature osteoclasts [13,14]. RANKL, as a member of the tumor necrosis factor (TNF) superfamily, binds to the RANK on osteoclast precursors to stimulate their differentiation and fusion into mature osteoclasts [15]. After treatment with RANKL, mouse RAW 264.7 cells have been shown to express high levels of osteoclast-related genes, including TRAP, nuclear factor of activated T-cells, cytoplasmic 1 (NFATc1), Cathepsin K (CatK), and Fra-2 [13,16]. The orchestrated process of osteoclast differentiation mainly involves rearrangement of the cytoskeleton, changes in the organelle types, and the degradation and renewal of intracellular proteins [17,18,19].

Flavonoids are the most common plant polyphenols and are present in the roots and leaves of plants and in many foods, such as green vegetables, fruits, soybean oils, red wine, and tea [12,20]. Recent research has focused on their antioxidant, anti-inflammatory, anticancer, and antibacterial activities [21]. More than 5000 different natural flavonoids have been described [22]. Kaempferol is a flavonoid compound produced from the stem and leaves of *Kaempferia galanga L*., which is used in traditional medicine, it has been associated with various biological functions, including anti-inflammation and antioxidant [23,24]. In addition, it has been shown that kaempferol promotes the differentiation and mineralization of osteoblasts and suppresses bone resorption and differentiation of osteoclasts [25,26,27,28]. However, the cellular mechanisms of flavonoid action on bone resorption have not been yet fully clarified.

Autophagy is a cell self-consumption process that is critical for cellular homeostasis; it is characterized by the sequestration of bulk cytoplasm, long-lived proteins, and organelle degradation [29]. Autophagy has pleiotropic functions that are involved in cell growth, survival, nutrient supply under starvation, defense against pathogens, and antigen presentation [30,31]. In cytokines, protein aggregation, damaged or surplus organelles, metabolic stress, hypoxia, and pathological conditions, autophagy is greatly increased, allowing the cell to degrade defected proteins and organelles to recycle macromolecular precursors, such as amino acids, fatty acids, and nucleotides [32]. Recent studies show that autophagy appears to be involved in the degradation of osteoclasts, osteoblasts, and osteocytes, potentially pointing to a new pathogenic mechanism for bone homeostasis and bone marrow diseases [33].

There are many studies dealing with the inhibitory effects of natural flavonoids in bone resorption [25,34,35]. However, until now, no studies have explained the relationship between the autophagic and inhibitory processes of osteoclastogenesis by natural flavonoids. The present study was undertaken to investigate the inhibitory effects of osteoclastogenesis through the autophagy process stimulated by kaempferol in RAW 264.7 cells.

## 2. Results

### 2.1. Inhibitory Effects of Kaempferol on RAW 264.7 Cells

**Figure 1:**
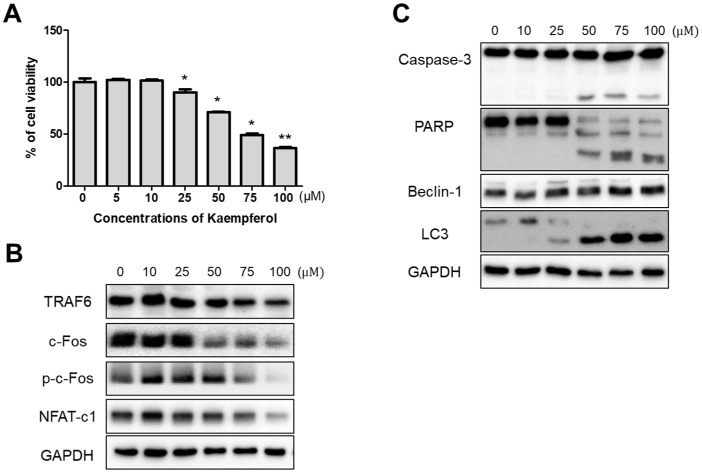
Cytotoxic effect of kaempferol on RAW 264.7 cells. (**A**) Cell viability was determined by MTT assay. 1 × 10^4^ cells/well of RAW 264.7 cell macrophages were seeded into a 96-well plate and incubated with various concentrations of kaempferol for 24 h. Kaempferol decreased cell viability of RAW 264.7 cells in a dose-dependent manner. Each value represents the mean of three independent experiments ± S.D. (*n* = 6). * *p* < 0.05 and ** *p* < 0.01 compared with the control (non-treated group); (**B**,**C**) Kaempferol inhibits osteoclastogenic factor and activates apoptosis and autophagy associated factors. Cells were treated with different concentrations of kaempferol (10–100 μM) for 24 h.

To investigate the cytotoxicity of kaempferol on RAW 264.7 cells, the cells were treated with various concentrations (5, 10, 25, 50, 75, and 100 μM) of kaempferol for 24 h and cytotoxicity was determined using an MTT assay. After 24 h of treatment, kaempferol did not affect cell viability at concentrations lower than 10 μM, but concentrations over 25 μM showed decreased cell viability in a dose-dependent manner (Figure 1A). To further evaluate the osteoclastogenic, apoptotic, and autophagic changes, we examined the inhibitory effect of kaempferol on RAW 264.7 cells via the use of a western blot assay. Osteoclastogenic factors, such as TRAF6, c-Fos, phopho-cFos, and NFAT-c1, showed that protein expression decreased with kaempferol treatments in concentrations over 50 μM. In addition, apoptosis related factors, such as caspase-3 and PARP, were activated to their cleaved forms, and autophagy induction proved the activation of beclin-1 expression and the conversion of LC3-I to LC3-II by kaempferol (Figure 1B,C). We found that kaempferol concentrations over 50 μM stimulated osteoclastogenesis, apoptosis, and autophagy associated factors in RAW 264.7 cells; therefore, we decided to use this concentration for the next experiments.

### 2.2. Kaempferol Inhibits RANKL-Induced Osteoclast Differentiation and Resorption in RAW 264.7 Cells

**Figure 2:**
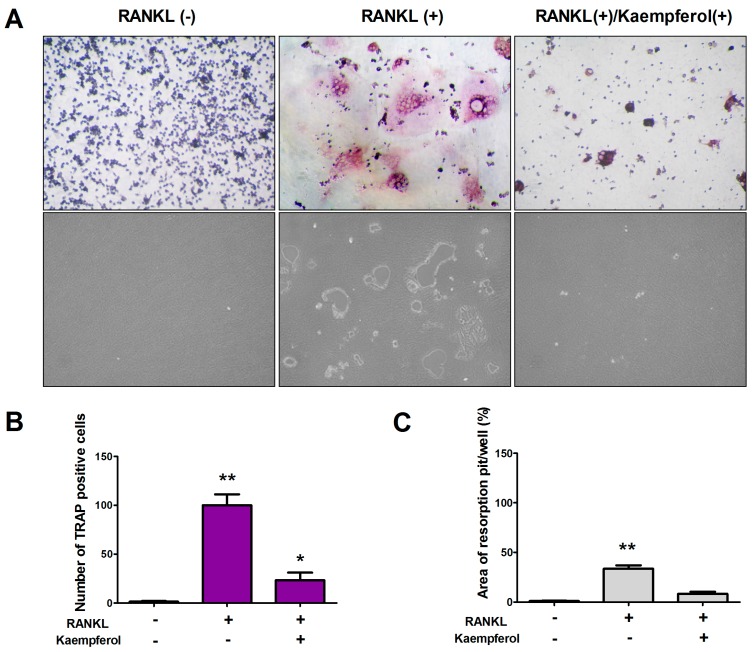
Inhibitory effects of kaempferol on RANKL-induced osteoclast differentiation. (**A**) RAW 264.7 cells were treated with 50 ng/mL RANKL then 50 μM kaempferol, respectively; to assess their inhibitory effects on osteoclast differentiation, TRAP staining was applied and TRAP-positive cells were visualized using light microphotography (upper panel). The inhibitory effect on bone resorption by kaempferol was assessed using a pit formation assay (lower panel); (**B**) The TRAP-positive multinucleated cells that contained three or more nuclei were counted; (**C**) The resorption areas were analyzed and the values expressed as the resorption pit area over the total area. Data are expressed as the mean ± S.D. (*n* = 5). * *p* < 0.05, ** *p* < 0.01 compared with the control group (RANKL(−)/Kaempferol(−)).

To examine the effect of kaempferol on osteoclast differentiation, staining was conducted for tartrate-resistant acid phosphatase (TRAP), an osteoclast marker enzyme. To induce osteoclast differentiation, RAW 264.7 cells were grown in osteoclast differentiation media with or without RANKL (50 ng/mL) for 9 days in 24-well plates. RANKL alone induced the formation of numerous multinucleated TRAP positive cells, but kaempferol (50 μM) inhibited RANKL-stimulated osteoclast-like cell formation in RAW 264.7 cells (Figure 2A,B). Kaempferol inhibits the formation of multinucleated cells, which leads to bone resorption. Therefore, we conducted a pit formation assay to find that kaempferol suppresses bone resorption by mature osteoclast. We demonstrated that the resorption pit area formed by the RANKL-stimulated RAW 264.7 cells was reduced in the presence of kaempferol (50 μM). As shown in Figure 2C, extremely large resorption pits formed in RANKL-stimulated RAW 264.7 cells. However, kaempferol treatment inhibited the RANKL-induced formation of resorption pits.

These results indicate that kaempferol effectively inhibited both osteoclast differentiation and resorption in osteoclastogenesis. 

### 2.3. Inhibition of Osteoclastogenesis and Autophagy by Kaempferol 

**Figure 3:**
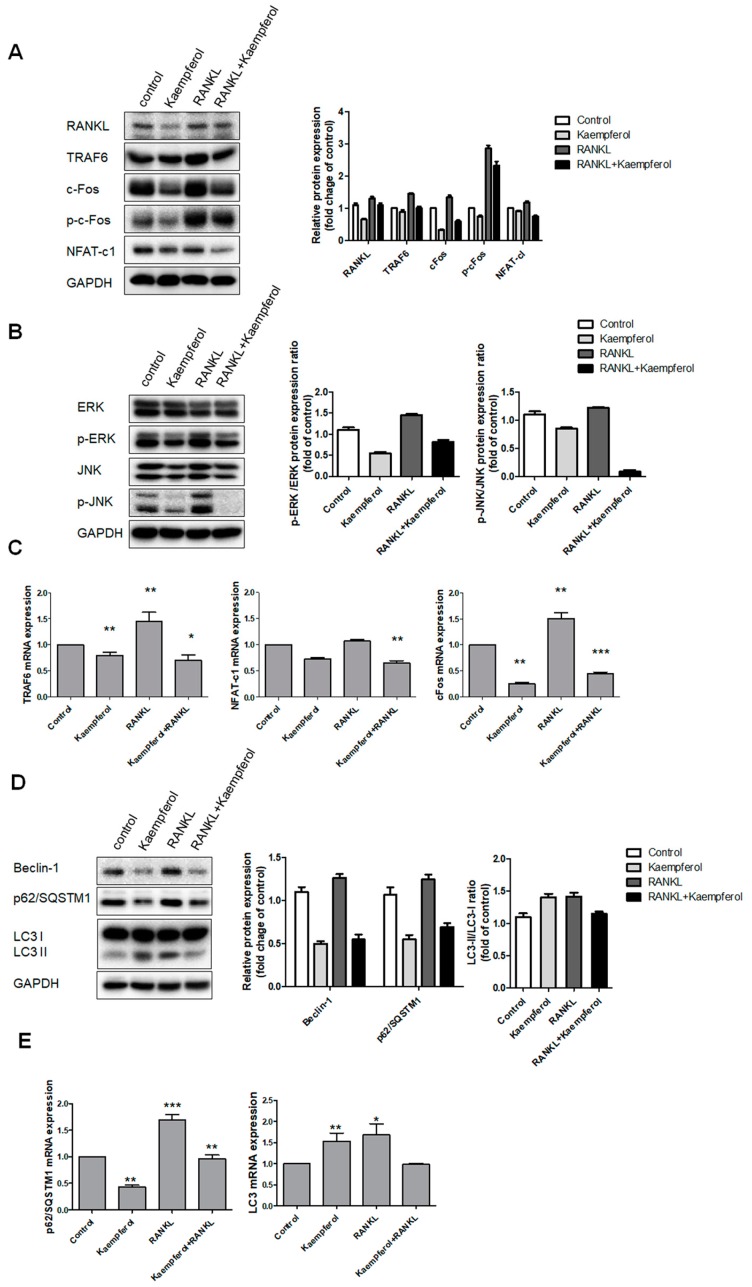
Effects of kaempferol on mRNA expression of osteoclastogenesis and autophagy related genes. RAW 264.7 cells with or without RANKL were treated with kaempferol (50 μM) and incubated for 2 h. Then, the protein samples were prepared for NFAT-c1 and ERK signaling (**A**). After treatment, the proteins RANKL, TRAF6, c-Fos, and NFAT-c1 were detected using western blot analysis. Kaempferol inhibited the NFAT-c1 signaling pathway; (**B**) Total and phosphorylated ERK and JNK were also inhibited by kaempferol. The mRNA expressions of the indicated genes (**A**–**C**) were analyzed using real-time PCR. Kaempferol inhibited NFAT-c1, TRAF6, and c-Fos. Each value represents the mean triplicate ± S.D. (*n* = 6). * *p* < 0 .05, ** *p* < 0.01, *** *p* < 0.001 compared with the control group. (**D**) Kaempferol (50 μM) inhibited autophagy related proteins such as beclin-1 and p62/SQSTM1 with or without RANKL (50 ng/mL), but LC3 was not suppressed. (**E**) The mRNA expressions of *p62/SQSTM1* and *LC3* were inhibited. Each value represents the mean triplicate ± S.D. (*n* = 6). * *p* < 0.05, ** *p* < 0.01, *** *p* < 0.001 compared with the control group.

To determine the effect of kaempferol on osteoclastogenesis, we investigated the protein and gene expression of *NFATc1*, *TRAF6*, *c-Fos, p62/SQSTM1*, and *LC3* through RANKL and autophagy signaling transduction using western blotting and real-time PCR. As shown in Figure 3A, RANKL increased the protein expression of RANKL, TRAF6, c-Fos, phosphor-cFos, and NFAT-c1. We next examined the effect of kaempferol on RANKL-induced ERK and JNK activation in RAW 264.7 cells. In the presence of 50 ng/mL RANKL, there was increased phosphorylation of the isoforms of ERK and JNK; in contrast, kaempferol inhibited these pathways (Figure 3B). These results suggest that although kaempferol can inactivate the ERK and JNK of MAP kinase, which are associated with the early RANKL-induced signaling pathways in RAW 264.7 cells, it inhibits the RANKL-mediated differentiation of the cells. To determine the mRNA expressions of osteoclastogenesis and autophagy related genes, we investigated the gene expression of *NFATc1, TRAF6, c-Fos, beclin-1, p62/SQSTM1*, and *LC3* using real-time PCR. RANKL increased the mRNA levels of *NFATc1, TRAF6, c-Fos, beclin-1*, and *p62/SQSTM1*, but kaempferol inhibited these factor (Figure 3C–E). However, *LC3* was not suppressed. These results indicate that kaempferol exerts suppressive effects on osteoclastogenesis and autophagy. 

### 2.4. Kaempferol-Inhibited Autophagy Was Related to p62/SQSTM1 Degradation

**Figure 4:**
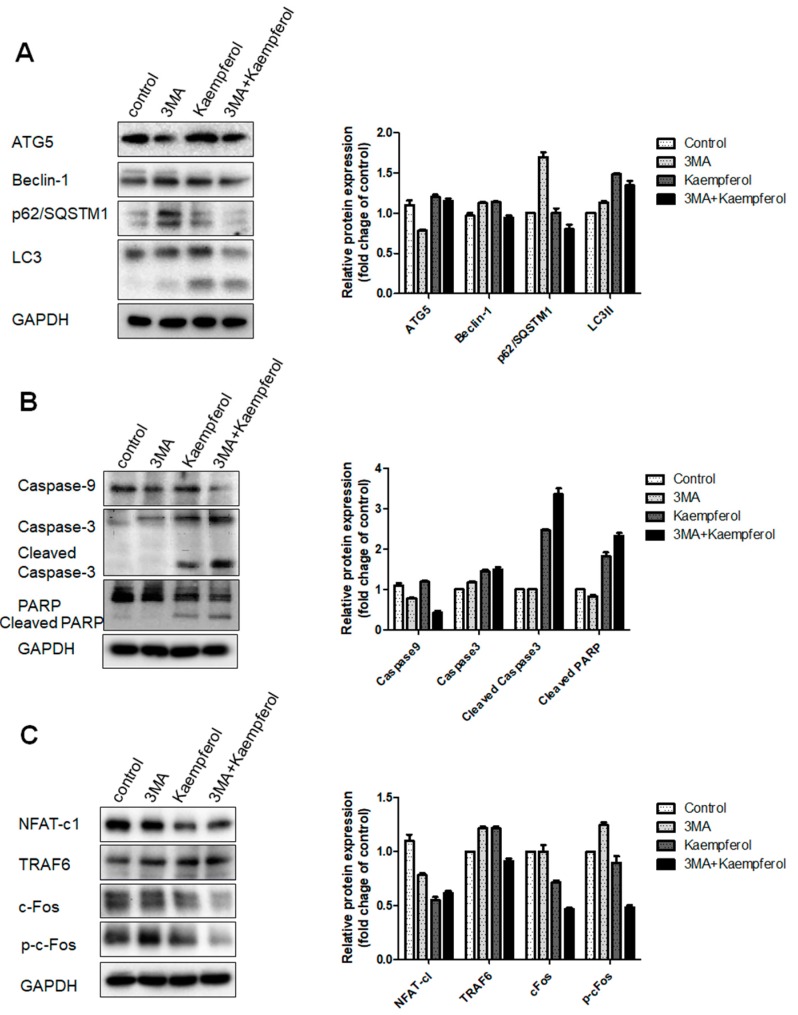

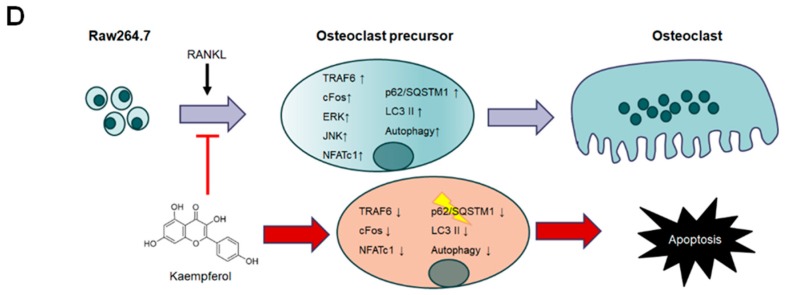
Kaempferol induced apoptotic cell death by degradation of p62/SQSTM1 in RAW 264.7 cells. Cells were pre-treated with 3-MA for 1 h and were then treated with kaempferol for 24 h. Autophagy (**A**); apoptosis (**B**); and osteoclastogenesis (**C**) related proteins were analyzed using western blotting; (**D**) Schematic representation for inhibition of osteoclast differentiation by kaempferol. Kaempferol inhibits autophagy through the suppression of p62/SQSTM1 and activates apoptosis.

To elucidate the kaempferol-inhibited autophagy associated with apoptosis in the pre-osteoclast RAW 264.7 cells, we used 3-methyladenine (3-MA), which prevents autophagy by blocking autophagosome formation via the inhibition of PI-3K. In order to investigate whether kaempferol-induced autophagy is involved in the cell death of the osteoclast precursor, we examined the effect of 3-MA on the expression of autophagy markers ATG5, beclin-1, p62/SQSTM1, and LC3, and the expression of apoptosis related proteins, caspase-9, -3, and PARP, as well as osteoclast related proteins, TRAF6, NFAT-c1, c-Fos, and phospho-cFos. Western blotting showed that 3-MA significantly inhibited the kaempferol-induced expression of autophagy markers such as ATG5, beclin-1, p62/SQSTM1, and LC3 (Figure 4A) and activated the cleaved forms of apoptosis related proteins, caspas-9, -3, and PARP (Figure 4B); there was no effect on the expression of osteoclast related proteins (Figure 4C). When only autophagy was suppressed, osteoclast and apoptosis related factors were not significantly affected. However, we found that after treatment with kaempferol, these factors inhibited autophagy and activated apoptosis. It would seem that p62/SQSTM1 plays a key role in this mechanism. Therefore, we presumed that kaempferol-inhibited autophagy and activated apoptosis through the degradation of p62/SQSTM1.

## 3. Discussion

In the present study, we proved for the first time that the kaempferol-inhibited autophagy suppresses osteoclastogenesis in RAW 264.7 cells.

Flavonoids have been found in food consumed in the human diet and have been used in traditional herbal medicine for thousands of years [36,37]. They are widely known to exert positive effects in the treatment of cancer, heart, neurodegenerative [38], and metabolic diseases [39]. Numerous recent studies report that flavonoids provide health benefits for the restoration of metabolic balance in the bone remodeling process by controlling osteoblast and osteoclast functions; it has been suggested that the consumption of certain flavonoids may contribute to bone health [27,40,41]. Kaempferol is a polyphenolic flavonoid extracted from many edible plants and traditional medicines, and has been shown to have strong antioxidant and anti-inflammatory properties [42,43]. In particular, it has been reported that kaempferol exerts inhibitory effects on the bone resorbing activity of osteoclasts [25] and an accelerator effect on the differentiation and mineralization of osteoblasts [26]. However, the inhibitory process of osteoclastogenesis by kaempferol is still unclear. Autophagy has well-known physiological and pathological roles in many organs [44,45]. Nevertheless, the physiological roles of autophagy in bone homeostasis and metabolic bone disease are also largely unknown [46]. The murine macrophage cell line RAW 264.7 was used in this study. RAW 264.7 cells express RANKL and very rapidly and readily differentiate into functional osteoclasts upon exposure to RANK [47]. Therefore, we focused on finding a relationship between kaempferol treatment and its potential effects on the suppression of osteoclastogenesis and the inhibition of autophagy in RAW 264.7 cells.

First, we determined the concentration of kaempferol that promoted osteoclast function by assessing the RAW 264.7 cells. Wattel et al. [25] reported that flavonoids, kaempferol, and quercetin increase osteoclastic programmed cell death in a dose-dependent manner. Our results showed that after 48 h of culture, the number of apoptotic osteoclasts in cultures treated with 50 μM of each flavonol increased approximately 3-fold as compared to the control culture. Autophagy undergo initiation, autophagosome double-membrane structure is enclose the cytoplasmic cargo protein or damaged small organelles [48]. When autophagosome formation is complete, fusion with the lysosome occurs to form an autolysome, which is degraded and reused as a nutrient [46,48]. The present study clearly showed that kaempferol reduced cell viability, inhibited osteoclasts, and induced apoptosis and autophagy related factors in a concentration-dependent manner in RAW 264.7 cells. These findings suggest that kaempferol augments autophagy and apoptosis in osteoclastogenesis. Osteoclastogenesis depends on the signal generated through RANK forming a complex with RANKL. The binding of RANKL to RANK results in a cascade of intracellular events, induces the key regulator of osteoclast differentiation, NFATc1, and activates NF-kB and MAPKs, including JNK [49,50]. Flavonoids have been shown to inhibit osteoclastic bone resorption and differentiate mature osteoclasts [25,51]. The present study showed that kaempferol inhibited the RANKL-induced differentiation of RAW 264.7 cells to osteoclasts by inhibiting the expression of TRAF6, NFAT-c1, and c-Fos. In particular, c-Fos and phospho-cFos were dramatically inhibited by kaempferol. Proto-oncogene c-Fos is isolated from murine osteosarcoma; c-Fos is known to be involved in the development of bones, teeth, and germ cells and in the central nervous system [52]. It also regulates MAPK family members, extracellular signal-regulated kinase (ERK), c-Jun N-terminal kinase (JNK), and p38 MAPK [53]. It has an essential role in osteoclast and macrophage differentiation from a common progenitor, and osteoclasts do not form in its absence [52,54]. Therefore, kaempferol clearly inhibited RANKL-induced osteoclastogenesis by inhibiting c-Fos expression and the formation of multinucleated cells.

Autophagy has pivotal roles in physiological and pathological processes in eukaryotic cells [55]. LC3, the first-identified mammalian protein, is generally used to monitor the process of autophagy. The lipidated form of LC3 transformed from LC3-I to LC3-II is considered to be an autophagosomal marker due to its localization and aggregation on autophagosomes [32,33]. In addition, p62/SQSTM1 becomes incorporated into autophagosomes and thus can also be used as an autophagy flux marker because of its characteristic accumulation accompanied by degradation [56]; its importance in bone cell activity is not yet known [33]. Recently, Li et al. [53] demonstrated that p62/SQSTM1 is an important bridge protein in RANKL-induced autophagy and osteoclastogenesis and that knockdown of p62/SQSTM1 inhibits osteoclastogenesis in RAW 264.7 cells. Our data clearly showed that kaempferol inhibited beclin-1 and p62/SQSTM1 and conversed LC3-I to LC3-II. These findings suggest that kaempferol suppresses osteoclast differentiation through p62/SQSTM1 degradation.

Blocking of ATG5, p62/SQSTM1, beclin-1 is often used because 3-MA can affect lysosomes independently in the autophagy process [57]. The conversion of LC3-I to LC3-II was inhibited by 3-MA, we found that 3-MA clearly reduced c-Fos and p62/SQSTM1 and activated the apoptosis related proteins, caspase-9, caspase-3, and PARP. These results indicate that kaempferol inhibited autophagy and that promoted apoptotic cell death and inhibited osteoclastogenesis in the RAW 264.7 cells.

The present study demonstrated kaempferol inhibits osteoclast differentiation and bone resorption through blocking autophagy. More biological studies would be necessary in which genes are controlled by kaempferol on autophagy and osteoclastogenic inhibiting process. However, kaempferol might help to target autophagy by emerging as a therapeutic alternative to treat metabolism diseases like osteoporosis. 

## 4. Materials and Methods

### 4.1. Reagents 

The following reagents were obtained commercially: Kaempferol, dimethyl sulfoxide (DMSO), aprotinin, leupeptin, phenylmethylsulfonyl fluoride (PMSF), and a TRAP staining kit were obtained from Sigma (St. Louis, MO, USA); recombinant RANKL was from R&D systems (Minneapolis, MN, USA); SuperSignal West Femto Enhanced Chemiluminescence western blotting detection reagent was from Pierce (Rockford, IL, USA). All other chemicals and reagents were purchased from Sigma unless otherwise specified. Primary antibodies against ERK, phospho-ERK, JNK, phospho-JNK, NFATc1, TRAF6, c-Fos, phospho-cFos, caspase-9, capsase-3, PARP, ATG5, beclin-1, p62/SQSTM1, LC3, and GAPDH were purchased from Cell Signaling Technology (Beverly, MA, USA). Secondary antibodies against mouse anti-rabbit IgG and rabbit anti-mouse IgG were purchased from Enzo Life Sciences (Farmingdale, NY, USA).

### 4.2. Cell Culture 

Murine monocytic macrophage RAW 264.7 cells were sourced from the Korean Cell Line Bank (KCLB; Seoul, Korea) and were maintained in Dulbecco Modified Eagle’s Medium (DMEM; Hyclone, Logan, UT, USA) with 10% heat-inactivated fetal bovine serum (FBS; Hyclone, Logan, UT, USA) at 37 °C in a 5% CO_2_ atmosphere. For osteoclast differentiation, cells were cultured in α-MEM medium (Gibco, Grand Island, NY, USA) supplemented with 10% FBS and 50 ng/mL recombinant mouse RANKL for 9 days.

### 4.3. MTT Assay

Cell viability was assessed by MTT assay. A total of 1 × 10^4^ cells were seeded and cultured in a 96-well plate, and then different concentrations of kaempferol were treated and incubated for 24 h at 37 °C in a 5% CO_2_ atmosphere, and an MTT solution (0.5 mg/mL) was added and incubated for 4 h. The absorbance was measured at 570 nm on an ELISA reader (Tecan, Männedorf, Switzerland).

### 4.4. Tartrate-Resistant Acid Phosphatase (TRAP) Staining

RAW 264.7 cells were suspended in α-MEM containing 10% FBS and plated at 5 × 10^4^ cells/well in a 24-well tissue culture plate with 50 ng/mL RANKL. The medium was replaced every 2 days. After 9 days, cells were fixed and stained using the TRAP activity staining kit according to the manufacturer’s instructions. TRAP-positive cells appeared dark red and TRAP-positive multinucleated cells with more than three nuclei were counted.

### 4.5. Osteoclast Pit Formation Assay

RAW 264.7 cells were suspended in α-MEM containing 10% FBS and plated at 5 × 10^4^ cells/well on an Osteo Assay plate (Corning, NY, USA) in the presence or absence of 50 ng/mL RANKL). The medium was replaced every 2 days. After 9 days of incubation, the osteoclasts were removed using a 5% sodium hypochlorite solution and the plate was washed twice with distilled water. The resorbed areas on the plates were captured with a digital camera attached to a microscope and analyzed by TSView 7 imaging software (Tucsen, Fuzhou, China).

### 4.6. Western Blot Analysis 

RAW 264.7 cells were seeded at 2 × 10^6^ in 100 mm culture dishes with medium and cultured for 1 day. At the end of each treatment of kaempferol, cell lysates were prepared. Protein concentration determination was performed with a protein assay kit (Bio-Rad, Budapest, Hungary). Proteins were loaded on a 10% SDS-PAGE gel, transferred to a polyvinylidene difluoride membrane (PVDF, Amersham GE Healthcare, Little Chalfont, UK), and reacted with each antibody. Then they were blotted with HRP-conjugated secondary antibody (1:5000). Immunoblotting with antibodies was performed using Super Signal West Femto Enhanced-Chemiluminescence Substrate and detected with an Alpha Imager HP (Alpha Innotech, Santa Clara, CA, USA). The total proteins were used for analysis of ERK, phospho-ERK, JNK, phospho-JNK, NFATc1, TRAF6, c-Fos, phospho-cFos, caspase-9, capsase-3, PARP, ATG5, beclin-1, p62/SQSTM1, LC3, and GAPDH.

### 4.7. RNA Isolation and Real-Time PCR

Total RNA was isolated from the cells using TRIzol reagent (Invitrogen Corp., Carlsbad, CA, USA). RNA (1 µg) was synthesized cDNA using the RevertAid First-Stand Synthesis System kit (Thermo Fisher Scientific, Pittsburgh, PA, USA) according to the manufacturer’s protocol. PCR Master Mix SYBR Green kit (Applied Biosystems, Warrington, UK) used for PCR amplification. Detection of fluorescent labeled reaction was performed using the ABI 7500 Real-Time PCR Detection System (Applied Biosystems, Foster City, CA, USA). TRAF6 (NM_009424), NFATc1 (NM_001164109), c-Fos (NM_010234), p62/SQSTM1 (NM_011018), LC3 (NM_025735), and GAPDH (NM_008084) messenger RNA expressions were quantified through a QuantiTec Primer Assay (Qiagen, Hilden, Germany).

### 4.8. Statistical Analysis

Statistical analysis data were expressed ± S.D. from at least three independent experiments. One-way ANOVA was used to analyze the data for cell viability, TRAP positivity, pit formation, and real-time PCR on a GraphPad Prism 5.0 (GraphPad Prism Software, San Diego, CA, USA). *p* values less than 0.05 were considered statistically significant.

## Abbreviations

BRONJ	Bisphosphonate related osteonecrosis of the jaws
BP	Bisphosphonates
TRAP	Tartrate-resistant acid phosphatase
MCSF	Macrophage colony-stimulating factor
RANK	Receptor activators of nuclear κB
RANKL	Receptor activators of nuclear κB ligand
TNF	Tumor necrosis factor
TRAF6	TNF receptor-associated factor 6
NFATc1	Nuclear factor of activated T-cells, cytoplasmic 1
CatK	Cathepsin K
DMSO	Dimethyl sulfoxide
PMSF	Phenylmethylsulfonyl fluoride
FBS	Fetal bovine serum
PVDF	Polyvinylidene difluoride membrane
MAPK	Mitogen-activated protein kinase
ERK	Extracellular signal-regulated kinase
JNK	c-Jun N-terminal kinase
PI-3K	Phosphatidylinositol 3-kinase
LC3	Microtubule-associated protein 1A/1B-light chain 3
ATG5	Autophagy protein 5
3-MA	3-Methyladenine
PARP	Poly ADP ribose polymerase
